# Study on human-SRL synchronized walking based on coupled impedance

**DOI:** 10.3389/fnbot.2023.1252947

**Published:** 2023-09-25

**Authors:** Zihao Liu, Kui Xiang, Wutong He, Xiang Gao, Yaling Peng, Muye Pang

**Affiliations:** School of Automation, Wuhan University of Technology, Wuhan, China

**Keywords:** supernumerary robotic limbs, human-machine coupling, synchronized walking, wheel-legged robotic limb, passive dynamic walking

## Abstract

**Introduction:**

Supernumerary robotic limbs (SRL) is a novel category of wearable robotics. Unlike prostheses (compensation for human limbs) and exoskeletons (augmentation of human limbs), SRL focuses on expanding human limbs and enhancing human activities, perception, and operation through the mutual collaboration of mechanical limbs and human limbs. The SRL of lower limbs are attached to the human waist, synchronized with the human walking in the forward direction, and can carry weight independently in the vertical direction.

**Methods:**

In order to enhance the synchronization performance of the human-machine system during walking and minimize interference with human gait, it is essential to investigate the coupling dynamics within the human-SRL system. To facilitate our research, this paper focuses on relatively ideal working conditions: level road surfaces, no additional weight-bearing on the SRL, and humans walking in a straight line without any turns. We build upon the passive dynamic walking theory and utilize the human-SRL system model established by MIT to develop a coupling system model. Through numerical simulations, we identify the optimal values for the stiffness and damping coefficients of the human-machine connection. Additionally, we have designed the wheel-legged SRL structure and constructed the SRL control system for experimental validation.

**Results:**

It is found that a better synchronization of the human-machine walking process can be achieved by configuring suitable spring and damping units in the human-machine connection part.

**Discussion:**

In this study, we explored the concept of SRL and its potential benefits for enhancing human motion, conducting simulations and experiments based on the coupled dynamics of human-SRL systems. The results indicate that by equipping the human-machine connection component with suitable spring and damping units, synchronization during the walking process can be improved.

## 1. Introduction

Human-machine collaboration has gained extensive application across diverse fields with the primary objective of elevating workplace safety, flexibility, and workload assistance. This collaboration can be broadly categorized into two forms: remote and wearable. In remote human-machine collaboration, including human-machine shared control (Luo et al., 2022), teleoperation techniques (Luo et al., 2020), interaction, and cooperation between humans and machines are facilitated through remote communication channels. This entails that humans and machines are not physically co-located but connected via networks or communication links. A quintessential example of remote collaboration is the operation of teleoperated robots, wherein human operators remotely control robots using controllers or computer terminals to execute tasks. Conversely, wearable human-machine collaboration involves machines or machine-assisted devices engaging in direct physical contact with human users to facilitate task completion. These devices, exemplified by exoskeletons or SRL (Gonzalez and Asada, 2018), are worn on the human body. This approach fosters a more intimate Human-machine collaboration, thereby fostering a more natural and seamless cooperation.

The SRL is a brand-new class of wearable robot, which extends human limbs by attaching to the waist or shoulder of a human. The structural forms of SRL include upper limbs, lower limbs, hands, and fingers, which improve human movement, perception, and manipulation by integrating and cooperating with human limbs. The SRL pushes the boundaries of human potential and improves limb functions by fusing human intelligence with mechanical force. A wide number of disciplines, including medicine, business, agriculture, politics, and daily life are all involved in the study of SRL (Parietti and Asada, 2016; Yang et al., 2021). It has many applications in numerous fields and offers technological support for human-machine collaboration and integration.

The research of the SRL originated from an upper limb designed by MIT's Asada team (Davenport et al., 2012), which was designed to provide a third and fourth arm to meet the needs of users in tight operating spaces, such as aircraft interior maintenance. A wearable robot secured on the shoulder of a human was created by Bonilla and Asada (2014). It was primarily used to assist users with tasks in the overhead workspace, such as installing a ceiling.

In recent years, as scholars from various countries continue to study in-depth, the SRL of lower limbs have gradually become a research hotspot in the scientific field. Parietti team (Parietti et al., 2015) designed a new wearable SRL that provides two additional legs to enhance the stability of the human-machine system and reduce the load on human leg joints. Gonzalez's team (Gonzalez and Asada, 2019) designed an extra robotic leg that assists in the transformation of human posture and is essentially a backpack with legs of its own, enabling the operator to walk around, climb stairs and crawl on the ground completely unhindered by their heavy payload. A team from Tsinghua University (Hao et al., 2020) created an additional robotic limb to help people walk while bearing weight. To help humans with weight-bearing transportation, Chenglong Fu and other researchers at Southern University of Science and Technology created a wheel-legged robotic limb (Leng et al., 2021). The system has a rigid robotic limb underneath the sprayer that transfers the sprayer's weight to the ground, reducing the weight carried by humans. Yang et al. presented a novel wearable robot, Centaur, designed as a load-bearing vehicle to assist human walking. They also proposed an interaction motion control strategy based on Human-machine collaboration forces to coordinate with human gait (Yang et al., 2022).

Currently, the research on the SRL primarily focuses on the upper limbs, and the lower limbs mainly adopt the leg structures. Although legged structures are more adaptable to terrain and environmental complexity, they also increase the complexity of structural design and control. In comparison, wheel-legged structures are relatively simpler. This paper takes the wheel-legged robotic limbs as a research topic and builds the SRL structure, establishes a human-machine system model, and conducts simulation in MATLAB. The stiffness and damping coefficient of the human-machine coupling is optimized, and the corresponding springs and dampers are selected as intermediate connectors between the human and the SRL. The coupling characteristics of the human-machine system are analyzed through experiments.

## 2. SRL structure design

### 2.1. Mechanical structure design

**Figure 1:**
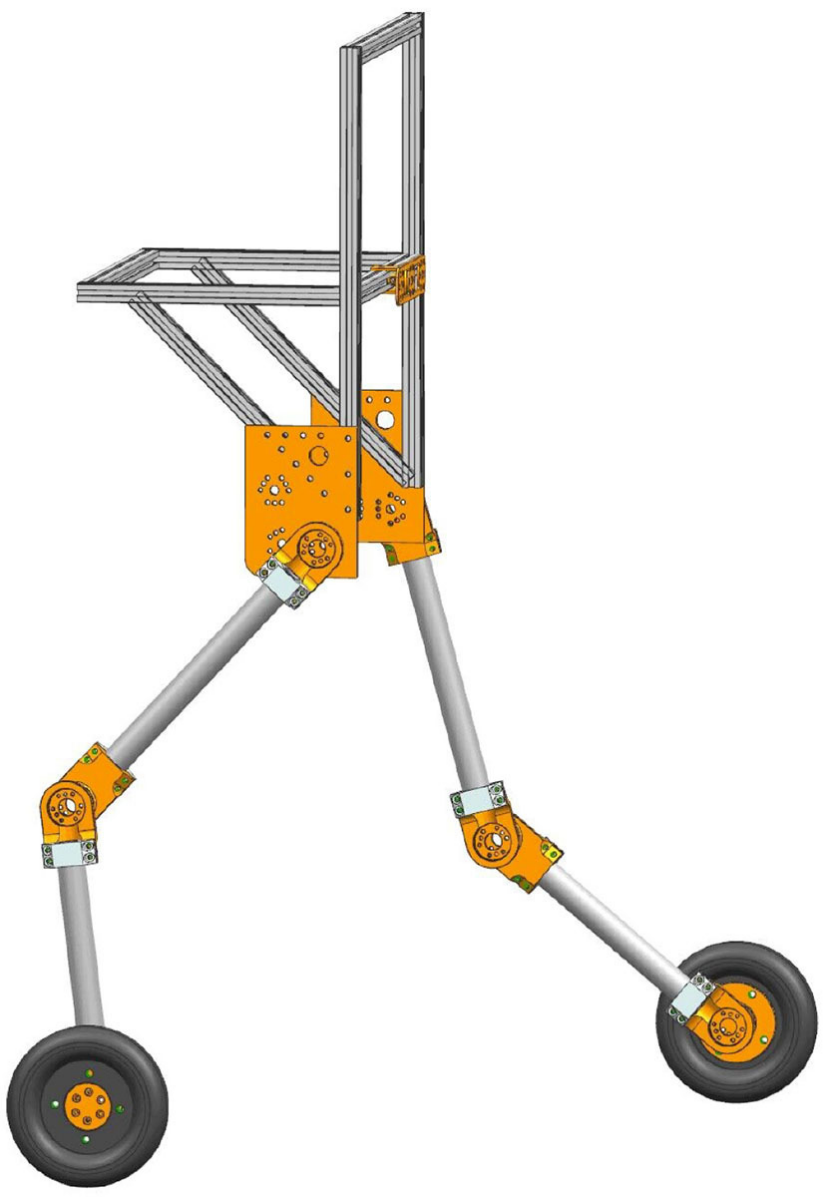
Design of the SRL structure.

In this paper, the major purpose of SRL is to follow a person when they walk without exerting excessive coupling force on them and to lighten their load. This SRL primarily comprises five parts, including the waist, two thighs, two lower legs, and a driving wheel with a rolling degree of freedom at the end of each lower leg. This paper does not release the hip and knee joint degrees of freedom as an initial study. The whole machine is very light, with a total weight of 9 kg. Figure 1 depicts the SRL structural layout.

**Figure 2:**
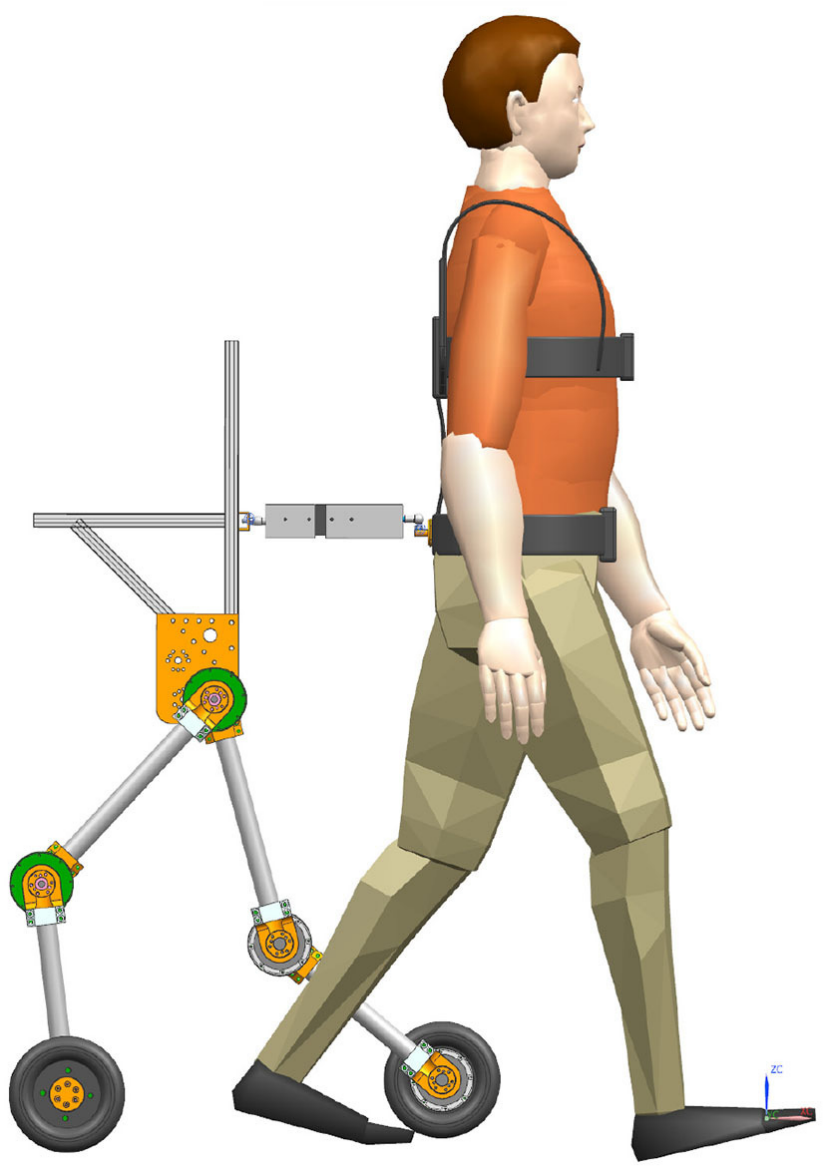
An illustrative representation of a user wearing the SRL.

**Figure 3:**
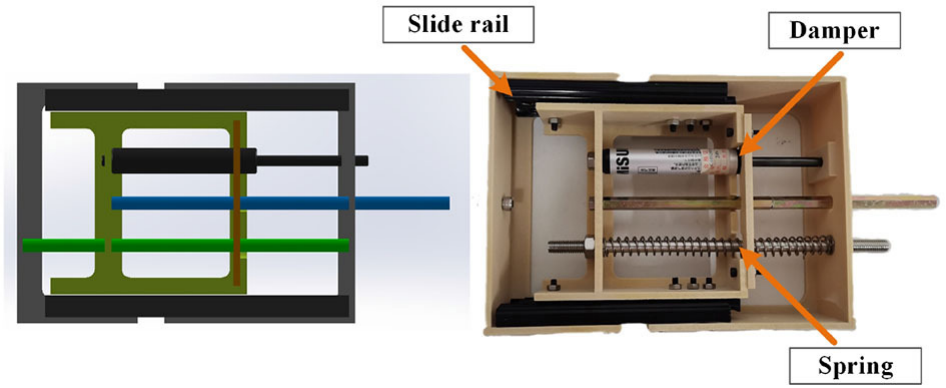
Mechanical design diagram and physical image of the coupler.

To wear the SRL, the wearer first utilizes a mountaineering harness to connect to the coupler, which is subsequently attached to the SRL, as depicted in Figure 2. The mechanical design diagram and physical image of the coupler are presented in Figure 3. The right end of the coupler is connected to the individual, while the left end is connected to the SRL, incorporating slide rails on both upper and lower ends. The mountaineering harness can be adjusted appropriately based on the wearer's body shape, providing a substantial load-bearing area that effectively reduces the wearer's burden and enhances overall comfort. It is essential to note that optimizing wearer comfort also contributes significantly to improving the control precision of the SRL.

### 2.2. Hardware design

**Figure 4:**
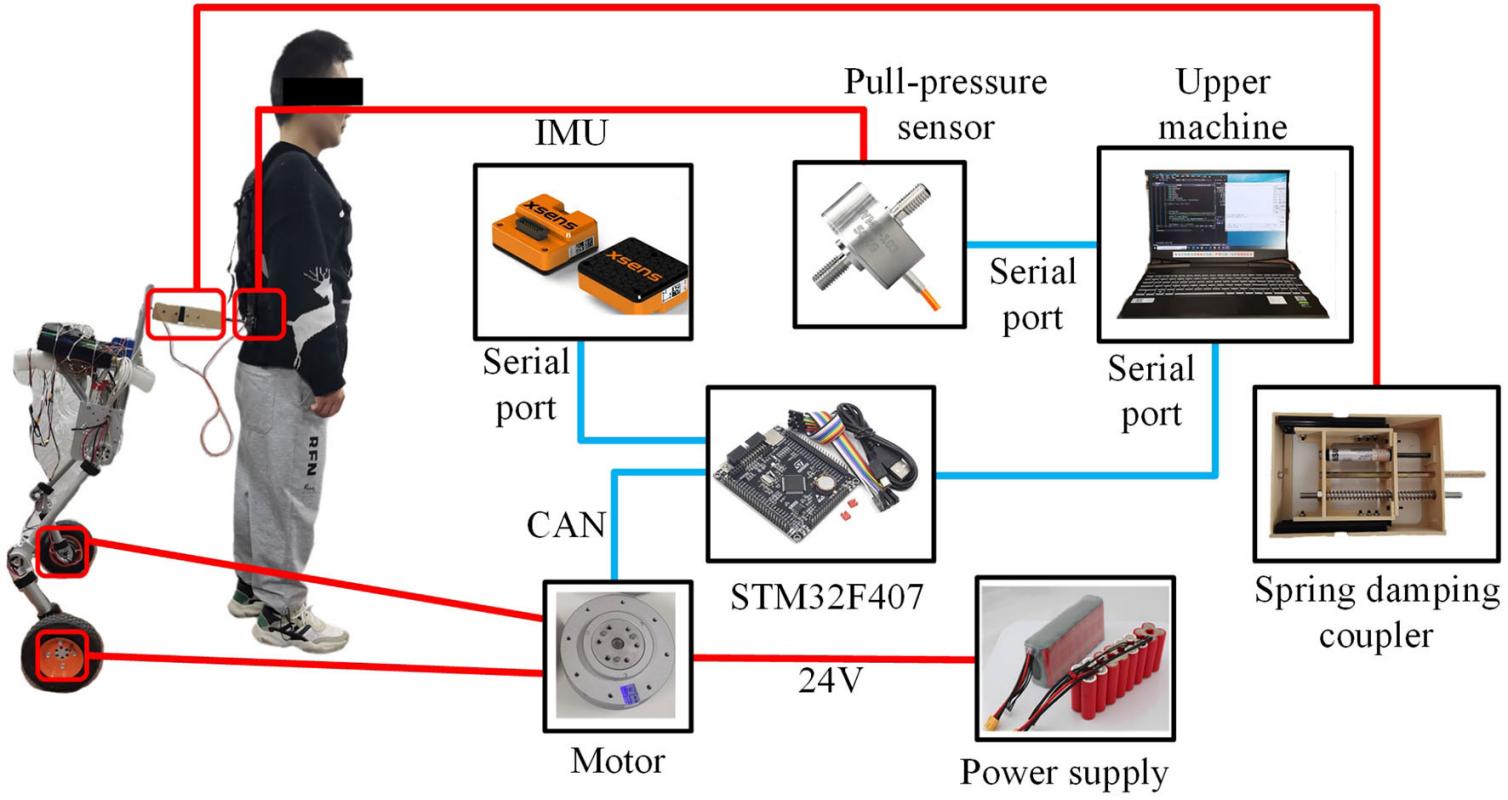
The hardware design of the SRL control system.

The hardware design of the SRL control system comprises essential components, including the Microcontroller Unit(MCU), measurement sensor, communication module, power supply, driver module, and coupler, as illustrated in Figure 4. Serving as the system's control center, the MCU, a STM32F407VET6 microcontroller from STMicroelectronics, assumes responsibility for communication, sensor data processing, and control program execution. The sensor section consists of the SRL posture sensor and the force sensor between the human and the SRL, which are integral to data collection. The posture sensor employs a series of inertial sensors, while the force sensor selected is the Dayang miniature tensile pressure DYMH-103 diaphragm sensor. For communication purposes, the system utilizes USART communication between the MCU and the inertial sensors, and CAN communication between the MCU and the drive wheel module. The power supply module ensures the provision of adequate power to the MCU and the drive system, ensuring smooth system operation. The ankle joint drive integrates a highly reliable quasi-direct drive module from YOBOTICS. This module employs three closed-loop control technologies (position outer loop, speed middle loop, and current inner loop) based on FOC, enabling effective regulation of motor position, speed, and torque. The design of the coupler considers the desired spring and damper characteristics.

### 2.3. Software design

**Figure 5:**
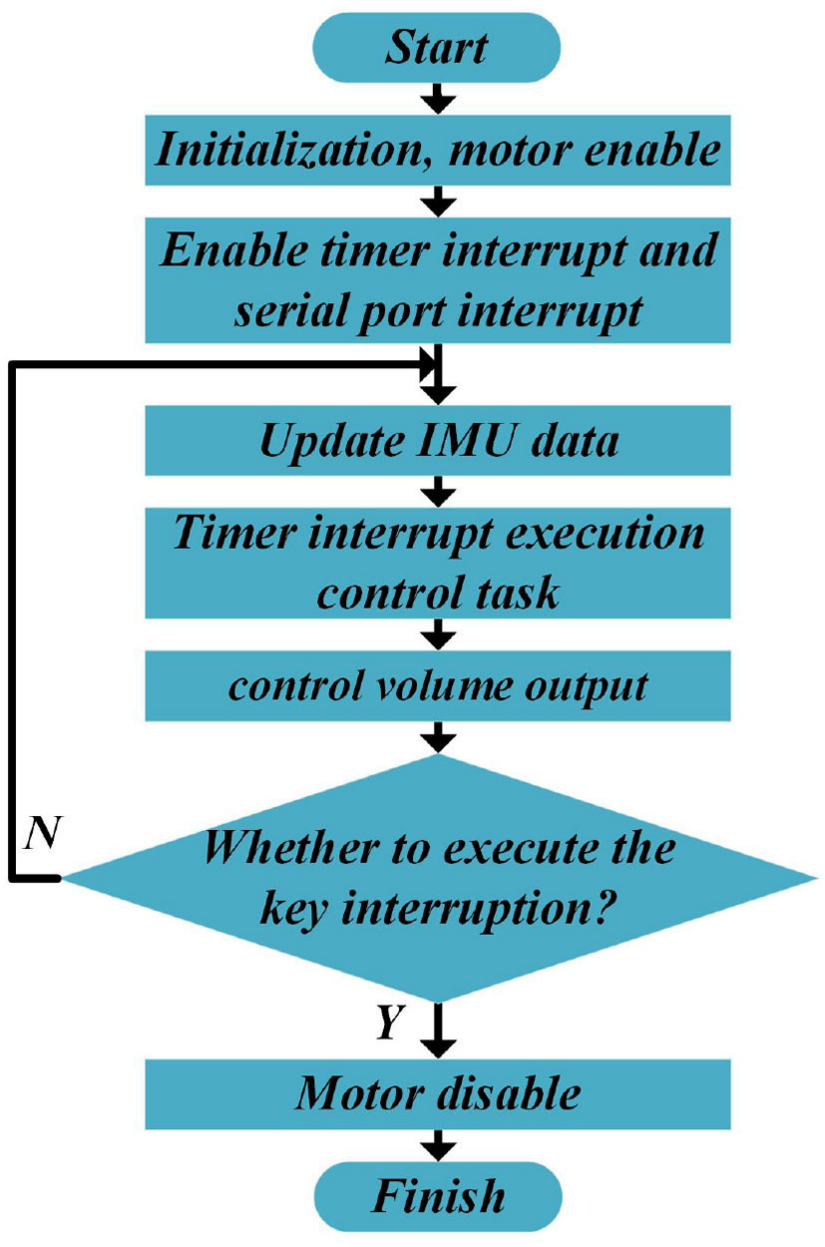
The control flowchart of the SRL.

After completing the design of the hardware system of the SRL, software programming is the key to the normal operation of the SRL control system. The control system aims to realize the self-driving of the SRL, the processing of sensor signals, and the implementation of motion control algorithms. Firstly, the system completes various initializations, enables the motor, turns on the timer and serial interrupt, and then enters the loop. Among them, the serial port interrupt is utilized to receive and parse the data sent by the IMU. The tasks executed in the timer interrupt function include calculating the required torque of the wheel motor in real time and sending it to the motor to realize torque control. Finally, determine whether the present task is complete, such as the case, execute the key interrupt to turn off the motor and conclude the control process, otherwise continue to execute the task in the interrupt function. The program flowchart is shown in Figure 5.

**Figure 6:**
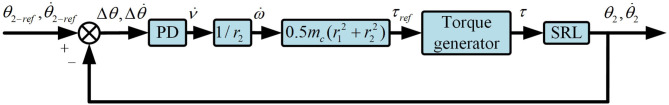
The diagram of the SRL motion control.

The control block diagram, as illustrated in Figure 6, delineates the motion control process of SRL. Herein, θ_2−*ref*_ and ${\dot{\theta }}_{2-ref}$ represent the angles and angular velocities required when SRL is positioned at its mechanical neutral point. At this juncture, SRL can maintain equilibrium without exerting tensile or compressive forces on the user.

By computing the difference between these values and measuring the actual tilt angle and angular velocity of the SRL, a PD control approach is employed to determine the desired acceleration of the wheel. Through a series of formula conversions, the motor drive torque for the wheel is ultimately derived, enabling precise motion control of the SRL.

## 3. Modeling and simulation

### 3.1. Modeling of the human-SRL system

**Figure 7:**
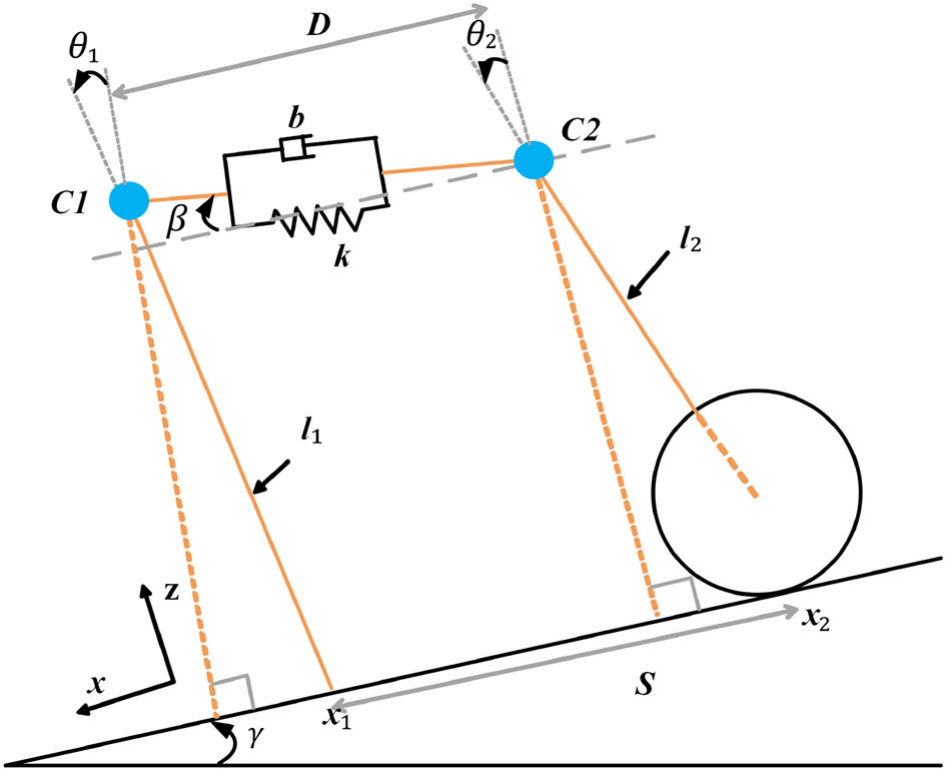
The model of the human-SRL system.

Gonzalez (Gonzalez and Asada, 2020) used a dynamic coupler consisting of a spring and a damper to connect two rimless wheels to enrich the dynamic interactions of the two bipedal systems, enabling the system to achieve the desired gait through intrinsic dynamics. Based on this, the paper proposes a design for the SRL as a wheel-legged structure rather than a legged one. Furthermore, a torque drive is applied to the SRL using PID control, enabling it to exhibit active motion. This design allows for the establishment of a new human-machine system dynamics model, as illustrated in Figure 7.

Given that human motion is predominantly powered by passive dynamic walking without any external force driving the model's movement, the introduction of slopes becomes essential (McGeer, 1990). This allows the model to emulate a stable gait similar to human walking, relying solely on its own gravity. Consequently, this paper focuses on the modeling of systems on slopes. The physical parameters utilized in this study are detailed in Table 1.

**Table 1:** Basic information of human-machine system model.

**Parameter**	**Definition**
*M*_*C*1_	Mass of the human body
*M*_*C*2_	Mass of the SRL
*m*_*C*_	Mass of the wheel
*l*_1_	Link length in human body models
*l*_2_	Length between the center of mass of the SRL model and the wheel center
θ_1_	Angle between the human body pendulum and the perpendicular line to the incline
θ_2_	Angle between the pendulum of the SRL and the perpendicular line to the incline
β	Angle between the coupler and the slope level
γ	Slope angle
*S*	Distance between the ground contact point of the human body model and the ground contact point of the SRL model
*S*_2_	Displacement of the SRL on the slope
σ	Fixed step length
α	Stride angle
τ	Torque generated by the wheel
*k*	Spring constant
*b*	Damping coefficient
*v*	Forward velocity of the wheel

In the current study, the human mass was 62 kg, while the mass of the SRL was 9 kg. The length of the linkage in the human model was precisely measured to be 0.9652 m.

For achieving a more precise emulation of human walking, the model incorporated an incline angle (γ) of 2°, and the step angle (α) was set to 15°. It is essential to highlight that certain parameters, like the spring factor (k) and damping factor (b), might necessitate fine-tuning based on specific circumstances to attain optimal gait performance. This attention to parameter adjustment is crucial for enhancing the model's ability to closely mimic human walking patterns.

During human walking, the height variation pattern of the center of mass exhibits a sinusoidal profile. When the SRL is used by an individual during walking, it also exhibits synchronized oscillations with the person's movements. To ensure the SRL achieves synchronized walking with human, it is imperative to equip the SRL with active motion capabilities. This objective can be accomplished by implementing torque control on the wheel motor. In this study, the acceleration ($\dot{v}$) of the wheel motor is assumed to be dependent on the inclination angle (θ_2_), inclination velocity (${\dot{\theta }}_{2}$), and slope inclination angle (γ) of the SRL, which can be expressed as follows:


(1) $$\begin{array}{l} \dot{v}=k_{p}\theta_{2}+k_{d}{\dot{\theta }}_{2}+g\sin\gamma \end{array}$$

where *k*_*p*_ and *k*_*d*_ are adjustable scale coefficients derived by field rectification. In this study, the final values of *k*_*p*_ and *k*_*d*_ are determined as 60 and 17, respectively. It is noteworthy that the direction of vector *v* aligns with the x-axis.

Conducting a comprehensive dynamics analysis of the human-machine system, the torque balance equation governing the system in this study can be expressed as follows:


(2) $$\begin{array}{l} M_{C1}l_{1}^{2}{\ddot{\theta }}_{1}=M_{c1}gl_{1}\sin\left(\theta_{1}+\gamma \right)-F_{c}l_{1}\left(\cos\theta_{1}\cos\beta -\sin\theta_{1}\sin\beta \right) \end{array}$$

(3) $$\begin{array}{l} M_{C2}l_{2}^{2}{\ddot{\theta }}_{2}=M_{C2}gl_{2}\sin\left(\theta_{2}+\gamma \right)+F_{c}l_{2}\left(\cos\theta_{2}\cos\beta -\sin\theta_{2}\sin\beta \right)-\tau \end{array}$$

where


(4) $$F_{c}=k\left(D-D_{0}\right)+b\dot{D}$$

Let the state vector be defined as follows:


(5) $$\begin{array}{l} x={\left[\begin{array}{ccccccc} \theta_{1} & {\dot{\theta }}_{1} & \theta_{2} & {\dot{\theta }}_{2} & D & S_{2} & v \end{array}\right]}^{T} \end{array}$$

Hence, the non-linear state equation of the human-machine system model can be derived as follows:


(6) $$\{\begin{array}{l} {\ddot{\theta }}_{1}=\frac{g}{l_{1}}\sin\left(\theta_{1}+\gamma \right)-\frac{F_{c}\cos\left(\theta_{1}+\beta \right)}{M_{C1}l_{1}}\\ \overset{..}{\theta_{2}}=\frac{g}{l_{2}}\sin\left(\theta_{2}+\gamma \right)+\frac{F_{c}\cos\left(\theta_{2}+\beta \right)}{M_{C2}l_{2}}-\frac{0.5m_{c}\left(r_{2}^{2}+r_{1}^{2}\right)\dot{v}}{M_{C2}l_{2}^{2}r_{2}}\\ \dot{D}=l_{1}\cos\left(\theta_{1}+\beta \right){\dot{\theta }}_{1}-l_{2}\cos\left(\theta_{2}+\beta \right){\dot{\theta }}_{2}-v\cos(\beta )\\ \dot{v}=k_{p}\theta_{2}+k_{d}{\dot{\theta }}_{2}+g\sin\gamma \\ S=n\sigma -S_{2} \end{array}$$

Where *n* represents the step count of the rimless wheel.

Before transitioning the swing leg to the support leg, discretization of the front pendulum dynamics is required. However, in the case of the established inverted pendulum model for the SRL, there is no explicit switching between the swing leg and the support leg, as the SRL wheel undergoes continuous motion on the slope. Therefore, separate dynamics treatment is not necessary for the SRL. The analysis of the step change is presented as follows for the front pendulum:

When *θ*_1_ > *α*:

An instantaneous change in angle occurs, *θ*_1+_ = −*α*.

The angular velocity also undergoes an instantaneous change, ${\dot{\theta }}_{1+}={\dot{\theta }}_{1-}\cos\left(2\alpha \right)$.

The step count of the rimless wheel is incremented by one, *n* = *n*+1.

### 3.2. Optimization of the human-machine coupling impedance parameters

In order to achieve improved coordination between the SRL and the human body, meticulous adjustments to the damping parameters within the coupler are also imperative. This is because an excessively high damping coefficient in the coupler can lead to the more pronounced transmission of asynchronous movements from the SRL to the human body, thereby severely impacting wearer comfort and synchronization performance. Conversely, if the damping coefficient is chosen to be too small, the coupler may excessively expand and contract due to minor movements in the SRL, consequently rendering the sensor data within the SRL unreliable.

Therefore, while aiming for smooth motion, it is essential that we strike a balance when selecting the stiffness coefficient (k) and damping coefficient (b) parameters, in order to achieve optimal comfort and synchronization performance.

Due to the stable behavior of the human-machine system resembling a limit cycle oscillator, its stability and characteristics are represented by a closed and continuous trajectory loop. The variation of the distance (D) between the human and the SRL from one oscillation period to the next is analyzed using the Poincare return map. The Poincare return map P() is an autonomous function that captures the simplified state of a trajectory from time k to time k+1.


(7) $$\begin{array}{l} x_{p}\left[k+1\right]=P\left(x_{p}\left[k\right]\right) \end{array}$$

The function P() can be linearized as


(8) $$\begin{array}{l} x_{p}\left[k+1\right]=\frac{dP\left(x_{p}\left[k\right]\right)}{dx_{p}}x_{p}\left[k\right]=Ax_{p}\left[k\right] \end{array}$$

The eigenvalues (λ_*i*_) of matrix A represent the dynamic response of the system. If |λ_*i*_| < 1, for *i*∈[1:*n*−1], it indicates that during the system's state transition process, the eigenvalues will gradually decay, and divergence phenomena will not occur. Therefore, the discrete system is stable. By selecting the upright position of the human as the Poincare section, where θ_1_ = 0°, the distance (D) between the human and the SRL in the final desired state is close to the initial length, indicating that the spring and damping elements are in their natural state.

The Poincare return map can be intractable to solve, but the linear matrix A can be obtained numerically using data collected from simulated trajectories (Smith and Berkemeier, 1997). The eigenvalues of matrix A represent the convergence rate of the system. By performing this analysis for different system parameters and comparing their eigenvalues, the parameters that lead to the fastest convergence rate can be selected.

The model's differential equations were solved using the ODE45 function in MATLAB. A total of 144 pairs of different values were chosen as the initial values for the state variables, with an initial distance of 0.5 m between the human and the SRL and a slope angle (λ) of 2°. The spring coefficient ranged from 50 to 1,000 N/m with an interval of 50, and the damping coefficient ranged from 10 to 1,000 Ns/m with an interval of 10. Using the same initial conditions, a set of trajectories were generated for each [k, b] pair. Among these trajectory sets, only those with the maximum eigenvalue of matrix A less than 1 were considered valid sets. Therefore, 56 sets of [k, b] pairs that met the criteria were obtained through simulations. Each [k, b] pair corresponded to a unique matrix A.

**Figure 8:**
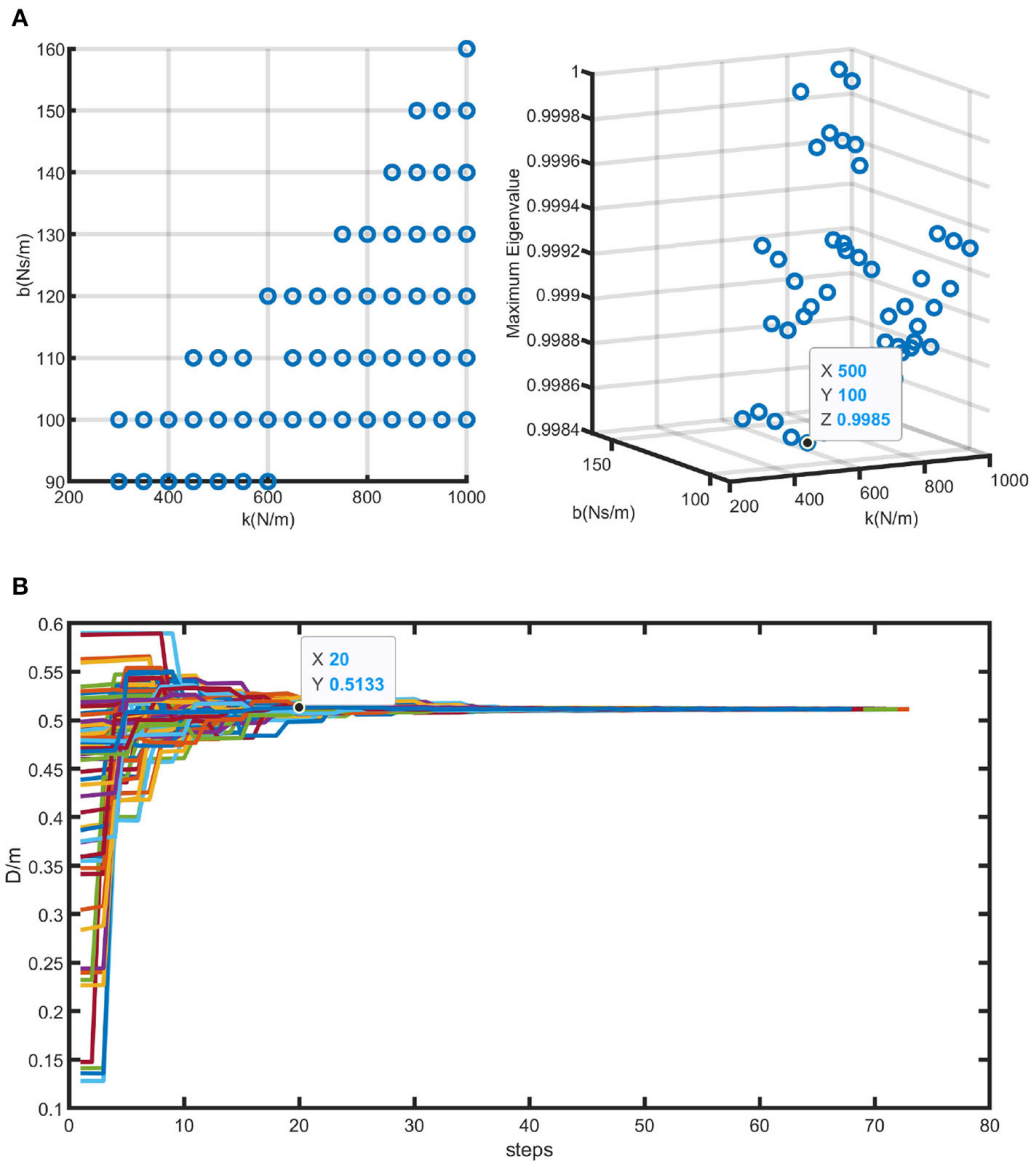
Simulation results of human-SRL synchronized walking. **(A)** Illustrates all the effective [k, b] pairs solved using the method proposed in this study, along with the corresponding eigenvalues of matrix A. The parameters leading to the fastest convergence are highlighted within the graph. **(B)** Illustrates the simulated results of human-SRL synchronized walking using the optimal parameters obtained from **(A)**.

Given that the system under consideration in this paper is discrete, a stability threshold of 1 is established. Consequently, as the eigenvalues move away from 1 while still being less than 1, the system's response becomes faster. Among the valid [k, b] parameter pairs, the pair with the smallest discrete eigenvalue was selected, with *k* = 500 N/m and *b* = 100 Ns/m. In this case, the eigenvalue is 0.9984. Figure 8A illustrates the valid [k, b] pairs and the maximum eigenvalue of matrix A for the parameter set with the fastest convergence rate.

The initial value of the distance (D) between the human and the SRL was set to 0.5 m. Figure 8B illustrates the convergence trajectory of D obtained using the optimal parameter set. The x-axis “steps” represents the number of walking steps in the simulation. From the graph, it can be observed that after approximately 20 steps, the value of D converges to 0.5 m, which is consistent with the initial state. This indicates that the human-machine system achieves synchronization during the walking process. Therefore, the optimized stiffness and damping coefficients, *k* = 500 N/m and *b* = 100 Ns/m serve as a theoretical basis for the selection of spring-damper units in the subsequent sections.

## 4. Experimental verification

### 4.1. Experimental platform construction

During the process of synchronization walking with human, the human-SRL system can be regarded as a hybrid quadruped system, combining human and robotic elements. Unlike quadruped systems where the four legs are coordinated and controlled by a central controller, the human-machine system is controlled by two independent control systems: the human brain controls the human part, while the SRL are controlled by their control system. Human gait cannot be directly controlled from the perspective of the SRL. Apart from adding sensors to the human body for SRL monitoring, the interaction between them is purely physical, relying on the inherent dynamic characteristics of both the human and the SRL to achieve synchronized walking. Physical couplers can be added between the two to indirectly affect the human. The human-machine system must take into account the uniqueness of the system: first, the bidirectional nature of dynamic interaction between the human and the SRL; second, the limited constraints on human gait imposed by physical coupling.

This paper presents the establishment of a spring-damper coupler to achieve synchronized walking in the human-machine system through intrinsic dynamics. The parameters of the spring and damper are selected based on the optimization results from previous simulations, with a chosen spring coefficient of 530 N/m and a damper stroke of 60 mm, resulting in a force of 20N.

During the process of human locomotion, the disparity in velocities between the human body and the SRL induces a sliding motion of the rail mechanism, thereby resulting in the compression of springs and dampers. This dynamic coupling effect between the SRL and the human body significantly enhances their dynamic interaction, thereby enriching their mutual dynamics.

**Figure 9:**
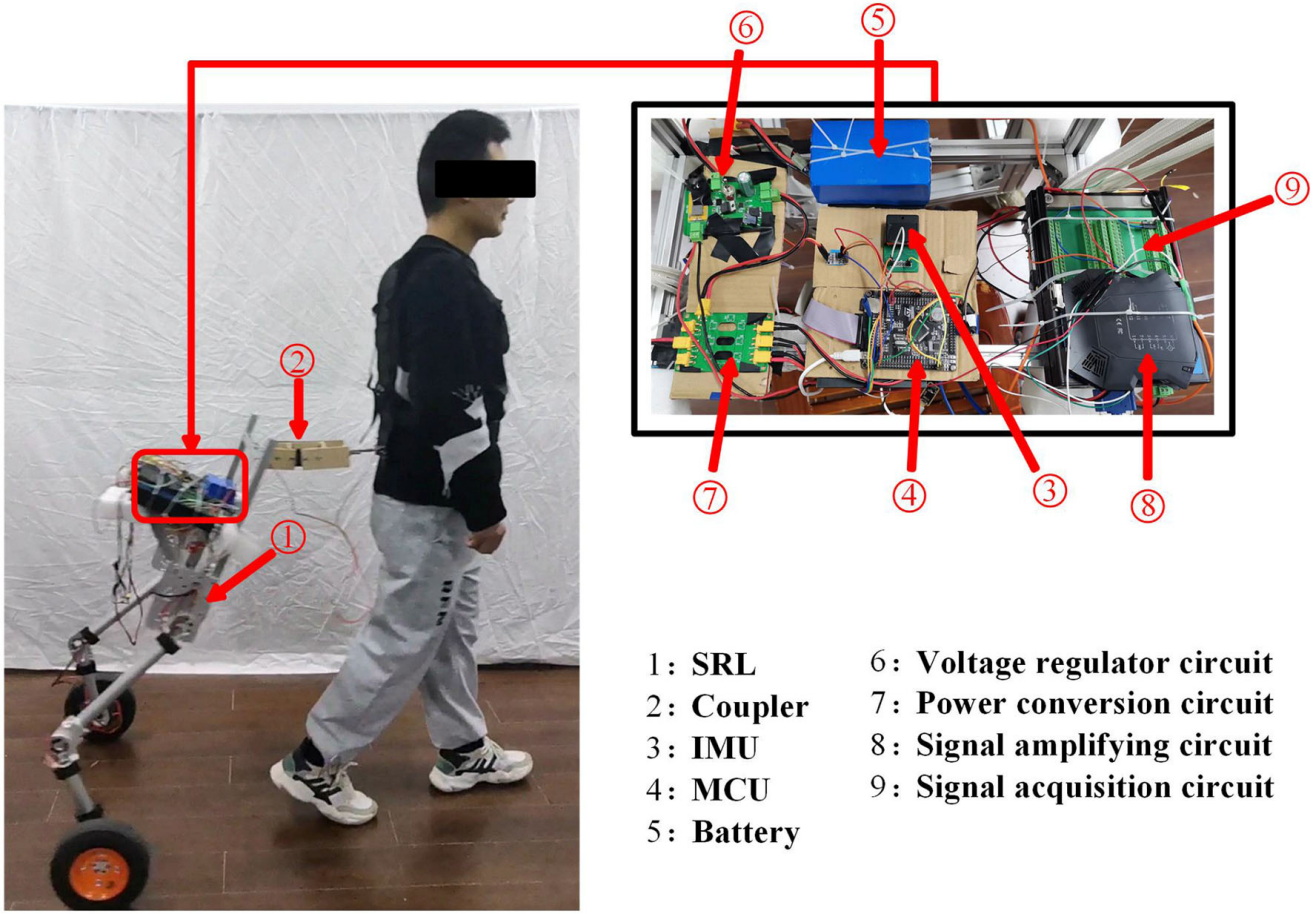
Experimental platform.

The experiment involved a total of six participants, aged between 22 and 26 years, with heights ranging from 165 to 180 cm. They had no history of movement disorders or lumbar diseases. The height of the SRL in this study was set at 100 cm to minimize the coupling forces between the participants and the SRL. To account for individual height differences and minimize errors, the software was used to set the mechanical mean value of the tilting angle of the SRL. The specific information of the participants is presented in Table 2. The experimental setup is shown in Figure 9, where the SRL was primarily constructed using aluminum profiles, resulting in a lightweight overall system. To investigate the dynamic characteristics of synchronized walking between the participants and the SRL, walking experiments were conducted in a 18-m-long corridor.

**Table 2:** Participant information.

**Subject number**	**Height (cm)**	**Lumbar connection height (cm)**	**Mechanical mean value of the SRL's tilt angle (deg)**
1	165	96	15
2	165	96	15
3	170	98	10
4	170	98	10
5	170	98	10
6	180	100	5

In the ideal scenario, external limbs should seamlessly coordinate with the wearer during synchronized walking, without inducing any discomfort. Previous studies have traditionally employed criteria such as force feedback, motion smoothness, and biomechanical analysis to assess the performance of external limbs in motion tracking. However, in this study, external limbs are exclusively linked to the human body via the waist region, thus centralizing their influence at the connection point. This is primarily due to the coupling forces arising from potential disparities in speed between the wearer and the SRL system. Consequently, this paper opts for the evaluation of external limb performance during human synchronized walking through the utilization of force feedback.

### 4.2. Experimental comparison of different coupling impedance parameters

**Figure 10:**
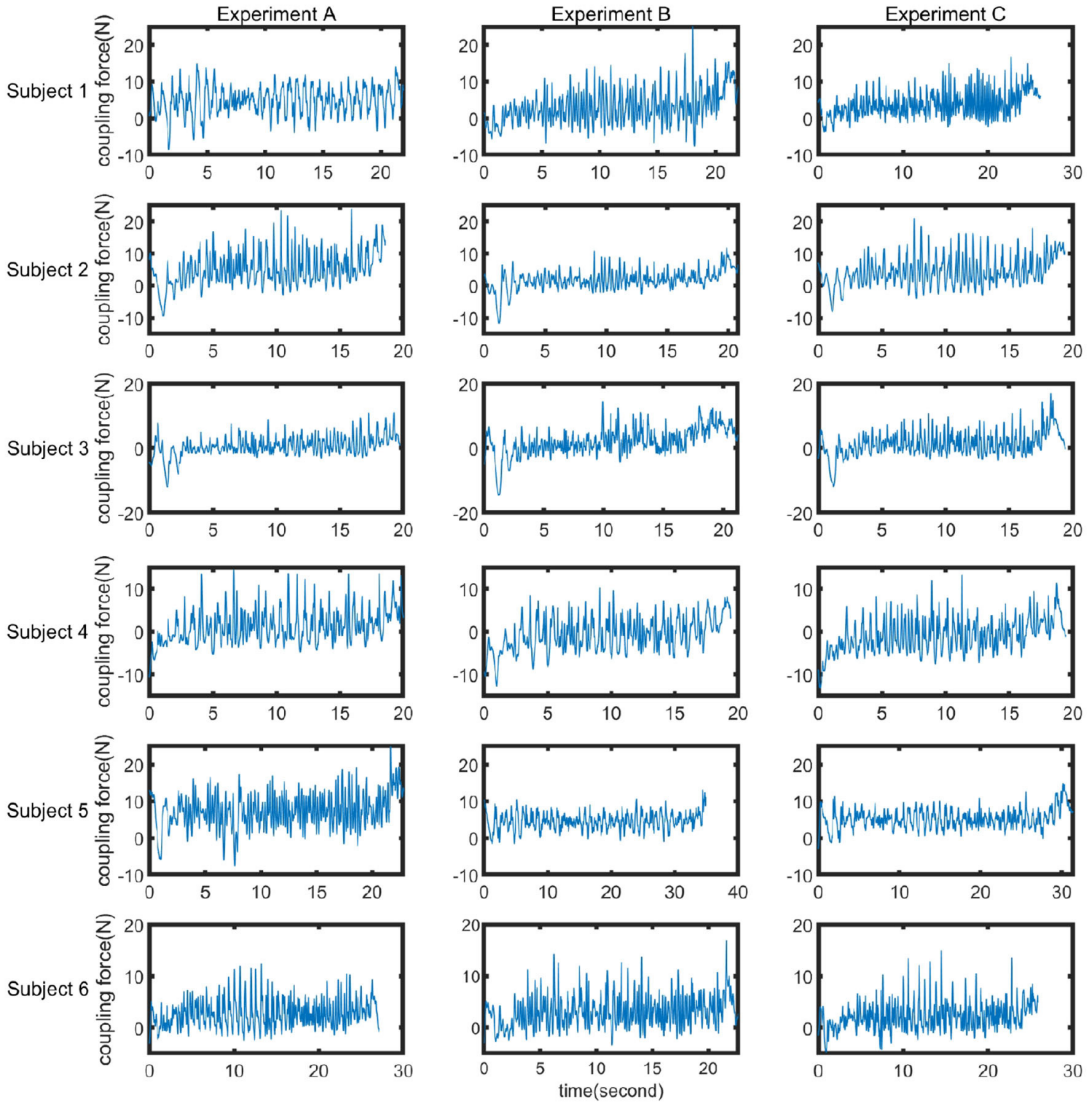
Comparison of coupling forces in experiments A, B, and C.

In order to investigate the impact of the spring and damper in the coupler on the dynamic characteristics of human-machine synchronized walking, three sets of experiments (A, B, and C) were conducted. In Experiment A, only the spring was included in the coupler. In Experiment B, only the damper was included. Experiment C involved both the spring and the damper in the coupler. Under these three experimental conditions, dynamic walking experiments were performed with the SRL. The average coupling force curves obtained from the three sets of experiments for the six participants are shown in Figure 10, and the corresponding mean values and variances are presented in Table 3. From Figure 10, it can be observed that at the end of each experiment, the coupling force exhibits a pulse waveform. This is due to the inertia of the SRL, which continues to move forward after the participant stops, resulting in a significant pushing force on the participant.

**Table 3:** Participant information.

**Subject number**	**Mean/ variance**	**Experiment A**	**Experiment B**	**Experiment C**
1	Mean	3.04	−2.68	3.76
	Variance	21.64	12.55	11.35
2	Mean	5.09	1.85	4.29
	Variance	26.41	9.15	20.82
3	Mean	0.45	1.96	1.23
	Variance	8.69	17.27	14.04
4	Mean	1.15	−0.75	−1.01
	Variance	14.83	15.58	15.49
5	Mean	7.34	4.60	5.33
	Variance	21.32	5.14	6.62
6	Mean	2.56	3.03	2.24
	Variance	6.39	9.12	6.89

To ensure minimal disruption to human ambulation caused by SRL, it is essential to maintain low values for both the mean and variance of the coupling force. This requirement stems from the fact that the mean reflects a persistent perturbation exerted by SRL across the entire human walking process, while the variance captures the magnitude of instantaneous perturbations introduced by SRL during various phases of human gait.

**Figure 11:**
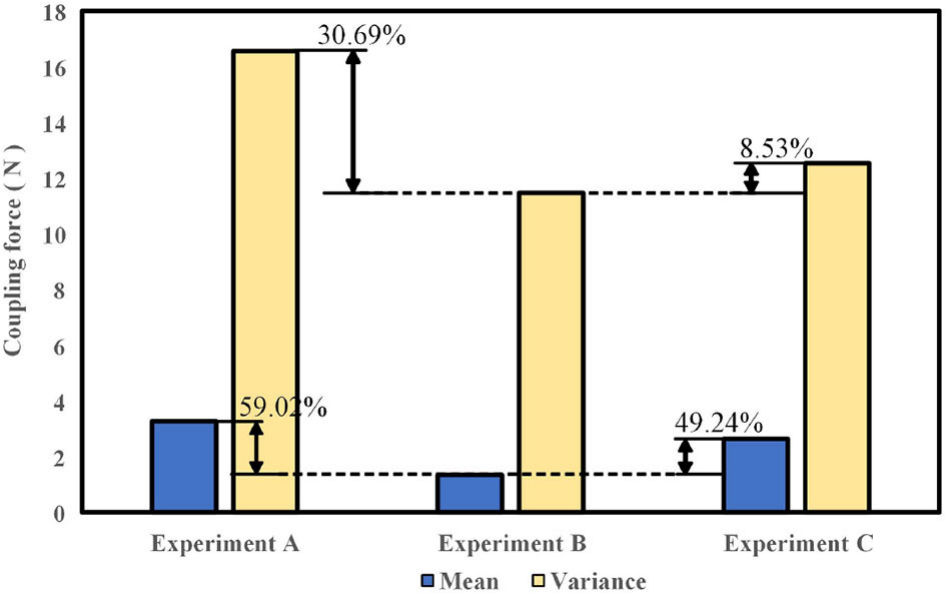
Average and variance of coupling forces under different coupling impedance parameters.

Considering the individual variations in walking habits among the six participants, the data were averaged for analysis in Experiments A, B, and C. As shown in Figure 11, the average coupling force values for the six individuals were determined to be 3.27 N, 1.34 N, and 2.64 N for Experiments A, B, and C, respectively. The average coupling force in Experiment B decreased by 59.02% and 49.24% compared to Experiments A and C, respectively. The average variances were 16.55 N, 11.47 N, and 12.54 N for Experiments A, B, and C, respectively. The variance in coupling force in Experiment B decreased by 30.69% and 8.53% compared to Experiments A and C, respectively. It can be concluded that under the conditions of Experiment A, the coupling force between the SRL and the participant was the highest, with an average value of 3.27 N, resulting in a pushing force on the participant. In Experiment B, the average coupling force was the lowest, indicating minimal interference from the SRL during human-machine synchronized walking, and the dynamic coupling characteristics of the human-machine system were optimal. The variance was also the smallest, indicating minimal fluctuations in the motion of the SRL during synchronized walking, resulting in smoother walking.

## 5. Discussion

**Figure 12:**
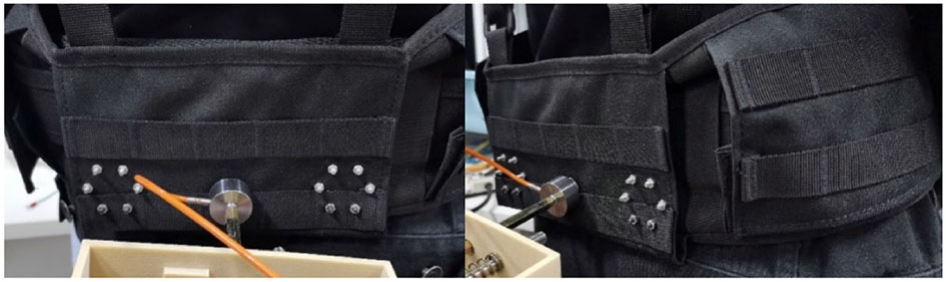
Schematic diagram of the connection at the human waist.

The experimental results reveal that the best performance in human-machine synchronized walking is achieved under the condition where only the damper is employed, which differs from the simulation results. Through analysis, three main reasons have been identified:

(1) The connection between the human and the SRL is not rigid but rather a soft connection facilitated by a mountaineering strap, as shown in Figure 12. The mountaineering strap itself possesses elasticity, and when combined with the elasticity of the spring, it increases the overall system's stiffness, thus influencing the experimental outcomes.

(2) The damper used in this study is not pure, instead, an air spring is utilized as a substitute. The air spring itself has a certain degree of elasticity, which also contributes to increased stiffness in the system, resulting in deviations between the actual human-machine system outcomes and the simulation results.

(3) The simulation utilizes a wheeled cart-wheel model to represent the human body, where the human body is modeled as a rigid pendulum. However, in reality, the human body possesses elasticity, and accurately measuring the rigidity of the human body is challenging, thereby influencing the experimental results.

To measure the stiffness of the damper and the mountaineering strap, the following procedure is carried out: First, the participant wears the mountaineering strap, and the distance from the participant's waist to the end of the coupling device is measured, yielding a value of 0.28 m. Then, a force of 20N is applied to stretch the coupling device, and the distance is remeasured to 0.33 m. From this, the combined stiffness of the mountaineering strap and damper is determined to be 400 N/m. Through experimental verification, it can be concluded that the aforementioned reasons are the primary causes of the discrepancies between the simulation and actual results.

In the future work, the following aspects need to be improved:

(1) The design of the wheel-legged SRL for synchronized walking in this study only considers the degree of freedom at the ankle joint, making it suitable only for walking on flat ground. However, for the human lower limbs, both the hip joint and the knee joint have multiple angles of freedom. Due to being an early-stage research product, the current structural design of this exoskeleton is relatively simple. For different tasks or working environments, additional degrees of freedom are required, along with further enhancement of torque control accuracy (Luo et al., 2023).

(2) The impedance parameters are optimized through simulation in this study, and a combination of spring-damper units with approximate coefficients is selected for the coupling device. There are certain differences between the physical parameters and the simulation results. If the physical system can be transformed into a virtual model for control, it would simplify the SRL structure and achieve better control performance. This is also a direction for future efforts.

(3) The current synchronization walking in this study requires several gait cycles to achieve. In the future, the tracking ability of the SRL to human movements will be improved by detecting the velocity of the human body's center of mass online.

## 6. Summary

The main focus of this paper is the system design and human-machine synchronized walking dynamics of an SRL. The research is centered around relatively ideal conditions: a flat surface, no load on the SRL, and straight-line walking without turning. Based on the theory of passive dynamic walking, a model of the human-machine system is developed, and optimal values for the stiffness and damping coefficients of the connection between the human and the SRL are obtained through simulation calculations. By selecting appropriate spring and damping units and designing a wheel-legged structure for the SRL, a control system is constructed to achieve better synchronization during human-machine walking. It is observed that there is a difference between the impedance parameter configuration of the connecting components and the simulation results, with the best synchronization achieved when only damping units are configured. This is attributed to the elasticity of both the human body itself and the harness at the human-machine connection. Existing synchronization walking requires several gait cycles to achieve. In the future, the tracking capability of the SRL for human motion will be improved by online detection of the human body's center of mass velocity.

## Data availability statement

The original contributions presented in the study are included in the article/supplementary material, further inquiries can be directed to the corresponding author.

## Ethics statement

Ethical approval was not required for the studies involving humans because this study only involves the wearing of supernumerary robotic limbs on the human body and does not involve in-depth research on the human body. The studies were conducted in accordance with the local legislation and institutional requirements. The participants provided their written informed consent to participate in this study.

## Author contributions

ZL, KX, and WH: conceptualization. ZL, KX, and MP: methodology and writing—review and editing. ZL, XG, and YP: writing—original draft preparation. All authors have read and agreed to the published version of the manuscript.
